# Yeast strains isolated from fermented beverage produce extracellular vesicles with anti-inflammatory effects

**DOI:** 10.1038/s41598-024-51370-7

**Published:** 2024-01-06

**Authors:** Stefano Nenciarini, Roberta Amoriello, Giovanni Bacci, Benedetta Cerasuolo, Monica Di Paola, Patrizia Nardini, Alessio Papini, Clara Ballerini, Duccio Cavalieri

**Affiliations:** 1 Department of Biology, University of Florence, Florence, Italy; 2 Department of Experimental and Clinical Medicine, University of Florence, Florence, Italy; 3 Department of Experimental and Clinical Medicine, University of Florence, Viale G. Pieraccini 6, 50139 Florence, Italy; 4 Department of Biology, University of Florence, Via Madonna del Piano 6, 50019 Sesto Fiorentino, Florence, Italy

**Keywords:** Applied microbiology, Food microbiology, Fungal host response, Fungal ecology, Antimicrobial responses, Chronic inflammation, Dendritic cells, Antigen processing and presentation, Drug delivery

## Abstract

Extracellular vesicles (EVs) are lipid-bilayered particles, containing various biomolecules, including nucleic acids, lipids, and proteins, released by cells from all the domains of life and performing multiple communication functions. Evidence suggests that the interaction between host immune cells and fungal EVs induces modulation of the immune system. Most of the studies on fungal EVs have been conducted in the context of fungal infections; therefore, there is a knowledge gap in what concerns the production of EVs by yeasts in other contexts rather than infection and that may affect human health. In this work, we characterized EVs obtained by *Saccharomyces cerevisiae* and *Pichia fermentans* strains isolated from a fermented milk product with probiotic properties. The immunomodulation abilities of EVs produced by these strains have been studied in vitro through immune assays after internalization from human monocyte-derived dendritic cells. Results showed a significant reduction in antigen presentation activity of dendritic cells treated with the fermented milk EVs. The small RNA fraction of EVs contained mainly yeast mRNA sequences, with a few molecular functions enriched in strains of two different species isolated from the fermented milk. Our results suggest that one of the mechanisms behind the anti-inflammatory properties of probiotic foods could be mediated by the interactions of human immune cells with yeast EVs.

## Introduction

Extracellular Vesicles (EVs) are one of the better-conserved mechanisms of intra- and inter-kingdom communication between organisms. Cell types from all the kingdoms of life produce and release membrane-bound vesicles to safely deliver a biomolecule cargo to a specific target^1^. The content of EVs can be very heterogeneous and ranges from proteins to lipids and nucleic acid material. Biogenesis, cargo loading, and releasing mechanisms differ among types of organisms and are still far from being fully understood^2,3^.

In fungi, EVs were described for the first time in 2007 in *Cryptococcus neoformans*^4^. The authors demonstrated that EVs were produced both in vitro and in vivo during infections and that they carried key virulence factors of *C. neoformans*. Since then, around twenty fungal species have been studied for their ability to produce EVs, including filamentous fungi, such as *Alternaria infectoria* and *Histoplasma capsulatum*, and yeasts, such as *Saccharomyces cerevisiae* and *Candida albicans*^5^. EVs are used by fungal species to modulate a series of different functions within the fungal community^6^, ranging from intercellular communication during biofilm formation to the regulation of intracellular proliferation and cell wall formation. Moreover, fungal EVs are known to mediate interactions with other fungi, bacteria, or superior host organisms, such as animals and plants^7^.

The interactions between fungi and their hosts through fungal EVs have been studied, especially during infections in mammalian cells, where fungal EVs can exacerbate or attenuate fungal infection by enhancing pathogenicity or modulating virulence strategies^8,9^. Pathogenic fungal EVs can penetrate host cells and modulate antimicrobial activities and host immune responses by activating the innate immune system and priming the activity of T cells through several mechanisms, including differential production of cytokines^5^.

There have also been a few studies regarding the immunomodulatory properties of EVs from human commensal yeasts, such as *Candida albicans*^10^ and *Malassezia sympodialis*^11^. Most of these studies focused on the capacity of EVs to trigger and activate phagocytes, such as dendritic cells (DCs), since they are mediators of adaptive immunity as well as key players in inflammatory processes and in the maintenance of homeostasis in body organs (especially the skin and intestine)^12^. It is known that DCs are involved in the reaction mechanisms of both pathogenic and commensal yeasts^13^. These antigen-presenting cells are indeed a crucial gateway for the initiation of immunity against foreign antigens since they contribute to homeostasis by sampling gut microbes and shaping immune responses in the lamina propria of the intestine^14^. The activation of DCs after antigen processing leads to differentiation and activation of T-helper (Th) cells by specific CD4^+^ effectors^15^. Thanks to this mechanism, DCs are considered the true mediators between the innate and adaptive immune systems and an important in vivo and in vitro target to study the organization of immune responses and tolerance to microbes^16^.

Many studies have identified RNA molecules in fungal EVs, both from commensal and pathogenic species, that could perform different functions in recipient cells^17^. Currently, these studies have focused mostly on small non-coding RNAs, i.e., molecules shorter than 200 nucleotides (nt)^18^, including microRNA (miRNA)-like sequences, small nucleolar RNAs (snoRNAs), small nuclear RNAs (snRNAs), and transfer RNA (tRNA) fragments, which seem to be differentially expressed in EVs and in host cells, suggesting the existence of an RNA sorting mechanism in EVs^19,20^. It is known that small RNAs can induce gene silencing through small RNA (sRNA)-mediated RNA interference (RNAi)^21^. In fungi, this mechanism has already been observed during interactions with plants^22^ and insects^23^. Some of the RNAs found in yeast EVs are known to have regulatory functions in host-microbe interactions. For instance, the well-studied miRNAs^24^ and the recently discovered tRNA fragments^25^ strengthen the hypothesis that these molecules are selectively loaded into EVs to mediate intra- and inter-species communications. To date, there have not been studies that show the effect of the RNA content of yeast non-pathogenic EVs on human immune cells.

It is well known that the gut microbiota composition can be shaped by diet, even in the relatively short-term^26^, and that foodborne *Saccharomyces* species with probiotic properties can modify the microbiota with beneficial effects on human health^27^. Following these assumptions and evidence on interactions between EVs and the host immune system, we focused on the characterization and immune properties of EVs produced by non-pathogenic foodborne yeasts, namely *Saccharomyces cerevisiae* and *Pichia fermentans*, commonly found in human guts^28^ and in fermented foods^29^. These strains have been isolated from traditional fermented beverages of the Yaghnob Valley (Tajikistan) and characterized in a previous work of our group^29^. Here, we found that human Monocyte-derived Dendritic Cells (Mo-DCs) showed significantly lower levels of activation in vitro in terms of antigen-presenting activity after exposure to fermented food-derived yeast EVs and that this effect, as well as the small RNA content, is strongly related to the yeast isolation source. Taken together, these results suggest that EVs could be suitable to shape host immunity, opening new possibilities for the therapeutic use of naturally produced EVs.

## Materials and methods

Yeast strains and growth conditions. The three yeast strains are part of the yeast laboratory collection of Prof. D. Cavalieri at the Department of Biology, University of Florence, Italy. The CL4 strain (Saccharomyces cerevisiae) and CL1 strain (Pichia fermentans) have been isolated from fermented goat milk of the Yaghnob Valley (Tajikistan), and they were characterized in our previous study 28. The ts9 strain (Saccharomyces cerevisiae) has been isolated from a Tuscan vineyard terroir. To enhance clarity, from now on the name of the species (Sc for Saccharomyces cerevisiae and Pf for Pichia fermentans) will be placed after the name of the strain as follows: CL4 (Sc), CL1 (Pf) and ts9 (Sc). Yeast strains were preserved in glycerol stocks and then inoculated overnight at 30 °C and 130 rpm in Yeast Extract–Peptone broth with glucose at 2% w/v (YPD) for microscopy observation and cell counting. For EVs isolation, overnight cells were counted on a Bürker chamber and then cultivated for 24 h at 30 °C and 130 rpm in YPD broth, starting with a concentration of 106 cells/mL. Yeast growths were carried out in Erlenmeyer flasks and evaluated every hour through cell counting and OD600 analysis on TECAN Infinite® 200 PRO (Tecan Trading AG, Switzerland; Supplementary material).

Extracellular vesicle isolation. EVs were isolated from yeast culture supernatants in YPD medium, as previously described^30^, with slight modifications. Briefly, cell-free culture supernatants were recovered by centrifugation for 15 min (4,000 × g, 8,000 × g, and 10,000 × g at 4 °C) to remove, respectively, cells, large debris, and small debris. The resultant supernatants were concentrated on Amicon ultrafiltration systems with a 100 kDa cut-off (Merck, Darmstadt, Germany). Concentrated supernatants were then ultracentrifuged for 1 h at 100,000 × g and 4 °C. After the ultracentrifugation, the supernatants were removed, and the pellets were washed in sterile PBS for another hour at 100,000 × g and 4 °C. After the washing step, the final pellets were resuspended in 100 µl of sterile PBS. The ultracentrifugation steps were carried out on the Optima MAX-XP Ultracentrifuge with a TLA 100.3 swinging bucket rotor (Beckman Coulter, Brea, California, USA). EV preparations were stored at − 80 °C.

Extracellular vesicles characterization. The characterization of EVs was carried out with Nanoparticle Tracking Analysis technology (NTA). EVs’ size distributions and concentrations were measured using the Nanosight NS300 platform (Malvern Panalytical, Malvern, Worcestershire, United Kingdom). The system was equipped with a sCMOS camera and a green laser and was running the NTA 3.4 analytical software package. The samples were analyzed at 100 X dilution in PBS with camera level 16 and detection threshold 4. EVs Zeta potentials were carried out with the same samples by Particle Metrix using a ZetaView® PMX 420 QUATT laser instrument (Particle Metrix, Meerbusch, Germany). The used software version was ZetaView 8.5.12 SP2 without CFR Part 11 functionality. Videos were recorded for each sample to visualize the vesicles.

Yeast microscopy. Yeast cells’ morphologies and concentrations were evaluated on a ZEISS® Primotech optical microscope equipped with an Axiocam 208 color camera and ZEN 3.3 software for image acquisition and processing (Zeiss, Oberkochen, Baden-Württemberg, Germany).

Transmission Electron Microscopy. The TEM analyses of yeast cells were carried out with a Philips 201 instrument operating at 80 KW (Philips, Amsterdam, Netherlands). Liquid cell cultures were prepared from glycerol stocks in 5 mL of YPD for 72 h, and after a washing step, the pellets were resuspended in 1 mL of glutaraldehyde 2.5% in phosphate buffer for at least 8 h (overnight) at 4 °C to let the glutaraldehyde fix the cells. The day after, cells were washed twice in a phosphate buffer with Liticase 28.5 U/mL to disrupt yeast cells’ walls. To fix membrane lipids, we added 1 mL of Osmium solution (OsO4) at 1% in the phosphate buffer. After 1 h, the cells were washed twice in 2 mL of phosphate buffer and dehydrated in an ascending alcohol series up to 100%. Then, we added 1 mL of propylene oxide and 1 mL of Spurr resin twice to embed the samples. After 1 h at 45 °C, the samples were washed to remove the supernatant and placed into wells (HistoMolds) to let the resin solidify at 70 °C overnight. Finally, the resultant resin blocks were cut into 80 nm slices with an Ultracut-E microtome (Reichert, Depew, New York, U.S.) and placed on formvar-coated grids to be observed at TEM. The TEM analyses of yeast EVs were carried out on a Jem 1010 Transmission Electron Microscope (Jeol, Tokyo, Japan) operating at 80 kV. Samples were prepared for transmission electron microscopy (TEM) using the negative staining procedure. In brief, the EVs pellets were fixed in Karnovsky’s fluid for 5 min at room temperature, centrifuged for 5 min at 11,000 g, and then rinsed and resuspended in 50 µL cacodylate buffer, 0.2 M. Aliquots of these suspensions were sedimented for 5 min at room temperature or 15 min at 37 °C on 300 mesh nickel carbon/formvar-coated grids. The Uranyless is used as a negative stain for both 5 RT and 15 min at 37 °C.

Immunological assays. All work with human study participants was approved by the Ethical Committees of the Azienda Ospedaliera Universitaria (AOU) Careggi (Ref. n. 87/10) and AOU Meyer Children’s Hospital (Ref. n. 103/2021), Florence, Italy. The research was carried out according to the principles set out in the Declaration of Helsinki 1964 and all subsequent revisions. Buffy coats were collected from healthy donors at the Transfusion Unit at Careggi University Hospital in Florence, Italy. The utilization of donor material, not destined to diagnostic standard procedures and registered with a traceable numeric code, was authorized by the Careggi Transfusion Unit. Informed consent was obtained from all subjects and/or their legal guardians.

Flow cytometry analysis of EVs endocytosis by Mo-DCs. At 7 days of differentiation, DCs maturation was assessed by a CyFlow Space cytometer (Sysmex Partec, Germany) by labeling cells with an anti-human CD11c PE antibody (clone 3.9; eBioscence, USA) for 20 min, RT, in the dark. Purified EVs were labeled with DiI stain (1,1’-dioctadecyl-3,3,3’,3’–tetramethylindocarbocyanine perchlorate) (Thermo Fisher Scientific, USA) as previously described^31^. Mature DCs (1 × 106) were cultured with or without EVs (approximately 1010 particles/mL), for 30 min at 37 °C, or at 4 °C as endocytosis negative control, in complete RPMI medium (RPMI 1640 with 10% fetal bovine serum, 1% penicillin–streptomycin, 1% sodium pyruvate, 1% l-glutamine, and 1% Hepes). After a gentle wash, EVs endocytosis was checked by flow cytometry evaluating DiI-labelled EVs Mean Fluorescence Intensity (MFI). Flow cytometry data acquisition was made by FloMax software (Sysmex Partec, Germany). MFI of 4 °C and 37 °C samples were compared by histogram overlaying of the DiI-EVs fluorescence within the CD11c + cell population. Events count from the histograms was normalized based on the maximum peak with FCS Express 6 Flow software (DeNovo software, USA).

Confocal microscopy analysis of EVs endocytosis by Mo-DCs. Endocytosis of EVs by DCs was further evaluated by confocal microscopy. Immediately after the endocytosis assay, cells were seeded on poly-L-lysine coated slides (approximately 5 × 105 cells/slide), fixed with 4% paraformaldehyde (PFA) for 15 min, RT, and washed three times with PBS. Cells were then permeabilized with a solution of PBS with 5% FBS and 0.3% Triton, for 30 min, RT. Permeabilized cells were labelled with Alexa Fluor 488 Phalloidin (Cell Signaling Technology, USA), staining cytoskeleton F-actin, for 15 min, RT, in the dark. Slides were then washed three times and closed with coverslip by using the ProLong™ Diamond Antifade Mountant with DAPI (Thermo Fisher Scientific, USA) to stain cell nuclei. Cells were dried, mounted onto glass slides, and examined with confocal microscopy using a Leica SP8 confocal microscope. A single composite image was obtained by superimposition of ten optical sections for each sample analyzed. Image size: 50 × 50 uM.

Dendritic cells isolation and stimulation. Mo-DCs were generated from human monocytes of healthy donors, as previously described^32^. Briefly, CD14 + monocytes were positively sorted by magnetic microbeads (Miltenyi Biotec, Bergisch Gladbach, North Rhine-Westphalia, Germany) and cultured for 6 days in a medium supplemented with GM-CSF (1000 units/mL, R&D Systems) and IL-4 (1000 units/mL, R&D Systems). At day 6 of culture, 105 immature DCs were activated by stimulation with either 1 μL/mL of LPS (Merck, Italy) or EVs (1:20 or 1:200 dilution) in a total volume of 100 μL. Then, the cells received the other stimulus. The incubation time of stimuli was 12 h for LPS and 4 or 24 h for EVs.

T cells isolation and Mixed Lymphocyte Reaction. A classic one-way Mixed Lymphocyte Reaction (MLR) with allogeneic T cells from healthy donors was performed to study the ability of human Mo-DCs to function as activators of T cells. Proliferation of T cells (Responder) is elicited through recognition of surface alloantigens (HLA-DR) on LPS-activated Mo-DCs (Stimulator). CD4 + T cells were negatively selected from peripheral blood mononuclear cells (PBMCs) of healthy donors using the T cell isolation kit II (Miltenyi Biotec, Bergisch Gladbach, North Rhine-Westphalia, Germany). The isolated T cells were used to test the antigen-presenting efficacy of 3 different allogeneic Mo-DCs concentrations (103, 5 × 103, and 104 cells in 200 µl) after activation with LPS (1 µg/mL) for 12 h and subsequent incubation with EVs for 4 or 24 h. Then, Mo-DCs were washed and resuspended in 100 µl of culture medium (RPMI 1640), where 105 T cells (in 100 µl of RPMI 1640) were added. For Mixed Lymphocyte Reaction (MLR), the incubation time was 5 days and the cells were plated in quadruplicate as previously described^32^. On day 5, the proliferative response was measured by 3H-thymidine (1 mCi/mL) in the incorporation test. 3H-thymidine was added for the last 8 h of culture. Plates were harvested with Tomtec Mach III (Hamden, Connecticut, U.S.) on glass fiber filters (PerkinElmer, Waltham, Massachusetts, U.S.), and 3H-thymidine uptake was measured by liquid scintillation in a Microbeta 1450 Trimux-counter (PerkinElmer, Waltham, Massachusetts, U.S.) and expressed as counts per minute (cpm).

Small RNA isolation. EVs were treated with Ribonuclease (RNAse) A (New England Biolabs, Ipswich, Massachusetts, U.S.) to degrade the RNA molecules not packed into vesicles. Then, EVs’ membranes were homogenized in 800 µl of Qiazol (Qiagen, Hilden, Germany) through multiple resuspension with sterile syringes (20-gauge) and needles. Small RNA-enriched fractions were isolated using the miRNeasy mini kit (Qiagen, Hilden, Germany) and then treated with the RNeasy MinElute cleanup kit (Qiagen, Hilden, Germany), according to the manufacturer’s protocol, to obtain small RNA-enriched fractions (< 200 nt).

Small RNA library preparation and sequencing. Library preparation and sequencing were performed by Novogene (Novogene, Beijing, China) on the Illumina Novaseq 6000 platform (Illumina, San Diego, California, U.S.), according to the standard pipeline for exosome sRNA sequencing. Illumina Novaseq 6000 SP was used and 20 M raw SE50 reads were obtained as output. Details about library and sequence preparation protocols are provided in Supplementary material.

Ribosomal sequence removal. Quality filtering of raw reads and removal of ribosomal sequences were the first steps for pre-processing of the RNA sequence dataset, to retain only the putative reads that don’t belong to ribosomes, merging supervised and unsupervised approaches (except for K-, where only the unsupervised approach could be possible since we could not assign it a custom ribosomal dataset). Ribosomal sequences were removed by using reference sequences downloaded from the Silva database^33^. All ribosomal sequences belonging to Pichia fermentans and Saccharomyces cerevisiae were downloaded from Silva and used to build Bowtie2 indexes (version 2.3)^34^. Bowtie2 was used for mapping reads against the respective ribosomal database, excluding only reads that fully align to ribosomes without taking into account clipped alignments. Samples exhibited a heterogeneous rate of ribosomal contamination, with Pichia being the species with the lowest rate. Since this effect could be due to the different number of sequences available in SILVA (Saccharomyces has more than 2300 sequences against the 320 of Pichia) we decided to also try an unsupervised method using Ribodetector^35^. Then, we combined the two approaches reported above by launching Ribodetector on the files aligned to the reference sequences.

Transcript quantification on human and yeast genomes. After quality control, the retained reads were mapped against S. cerevisiae and human reference genomes to understand, respectively, the types of RNA present in EVs and the sequences in the human genome that could be putative targets for RNAi activity or other RNA-induced regulation mechanisms by the small RNAs found in the EVs samples. Sequences were mapped against the latest human reference transcriptome (GRCh38.p13) available from GENCODE^36^. We quantified sRNA mapping on the human transcript by using Salmon (version 1.9)^37^ with selective alignment. Before mapping sequences to human transcripts, a list of decoy fragments was obtained from the human genome available in GENCODE and used to build Salmon index file. The latest release of Saccharomyces cerevisiae genome (S288C, dated: 2021/04/21) was downloaded from the “Saccharomyces Genome Database” (SGD) and indexed using salmon^38^. Salmon quantification was imported using the tximeta R package (version 1.17.1)^39^ and analyzed by using DESeq2 (version 1.38.1)^40^.

Statistics and data analysis. Count dispersion was fitted using “local” mode as it reported the lowest median absolute residual value. Counts were then transformed by using the “varianceStabilizingTransformation” function and scaled to mean equal zero and standard deviation equal one to report abundance values into a heatmap. Clustering was performed by transforming Kendall’s correlation. The final distance matrix was transformed into a cladogram by using the “unweighted pair group method with arithmetic mean” method (UPGMA).

Afterward, we analyzed the differential abundance of mapped reads both on human and S. cerevisiae genomes with different dispersion models (Figure S7 and S8, Tables S2 and S3). Differential abundance analysis was performed by comparing the three strains CL4 (Sc), CL1 (Pf), and ts9 (Sc) against the control K-. Fold changes were shrunk by using the apeglm package^41^ in combination with the lfcShrink function of DESeq2. Only genes with a *p* value lower than 0.05 and a Fold change higher than 1 when considered significant and used for subsequent analyses.

Enrichment analysis on highly abundant genes was performed by using Enrichr^42^. Genes were tested against all datasets available by using the enrichR package version 3.1. All acquisition and data analysis software used in the experimental procedures are specified in the text. Statistics and graphs for the RNA analyses have been generated using R Statistical Software (v4.2). Statistical analyses of immunological data were performed using GraphPad Prism 6 software. Results were expressed as means ± SEM and the performed statistical tests were one-way ANOVA followed by Tukey’s multiple comparison test. Statistical significance was for *p* values < 0.05.

## Results

### EVs from the foodborne yeasts *S. cerevisiae* and *P. fermentans* showed similar structural characteristics

**Figure 1:**
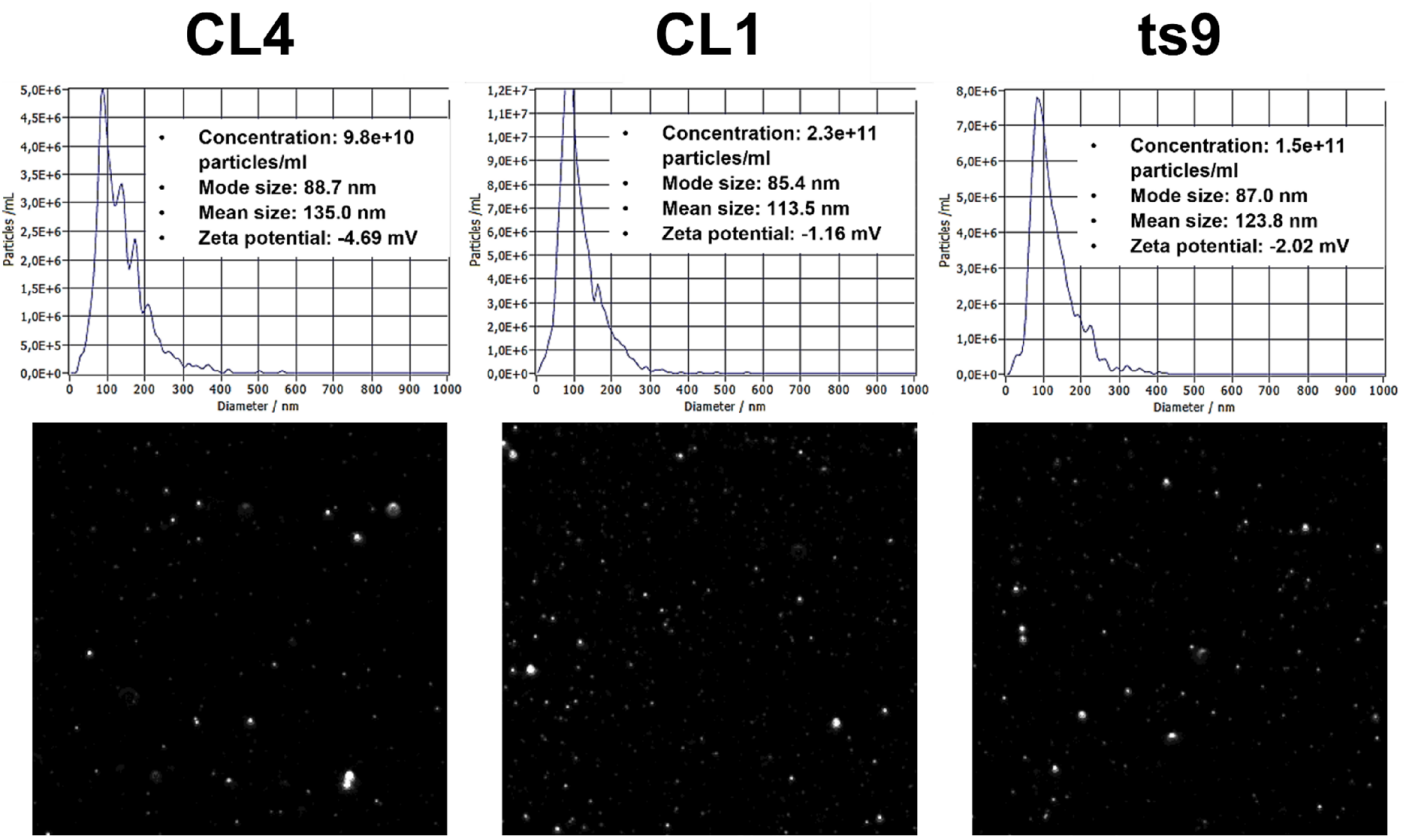
Characterization of Extracellular Vesicles (EVs) by Nanoparticle Tracking Analysis (NTA). EVs size distribution and concentration were measured with Nanosight NS300 (Malvern Panalytical, Malvern, Worcestershire, United Kingdom). Zeta potential and video frames were acquired from the same samples with ZetaView (Particle Metrix, Meerbusch, Germany). The analyzed yeast strains were CL4 (*Sc*), CL1 (*Pf*), and ts9 (*Sc*).

EVs were isolated from the supernatants of liquid cultures of three yeast strains: CL4 (*Saccharomyces cerevisiae*) and CL1 (*Pichia fermentans*), both isolated from the same milk-fermented beverage, and ts9 (*Saccharomyces cerevisiae*), an environmental strain from our laboratory collection (see details in the Materials and Methods section). EVs were then analyzed both by Nanoparticle Tracking Analysis (NTA) and Transmission Electron Microscopy (TEM). The NTA measurement showed similar size (mode size under 100 nm), concentration (ranging from 9.8 × 10^10^ to 2.3 × 10^11^ particles/mL), and Zeta potential (low negative values) among EVs from the different yeast strains (Fig. 1).

**Figure 2:**
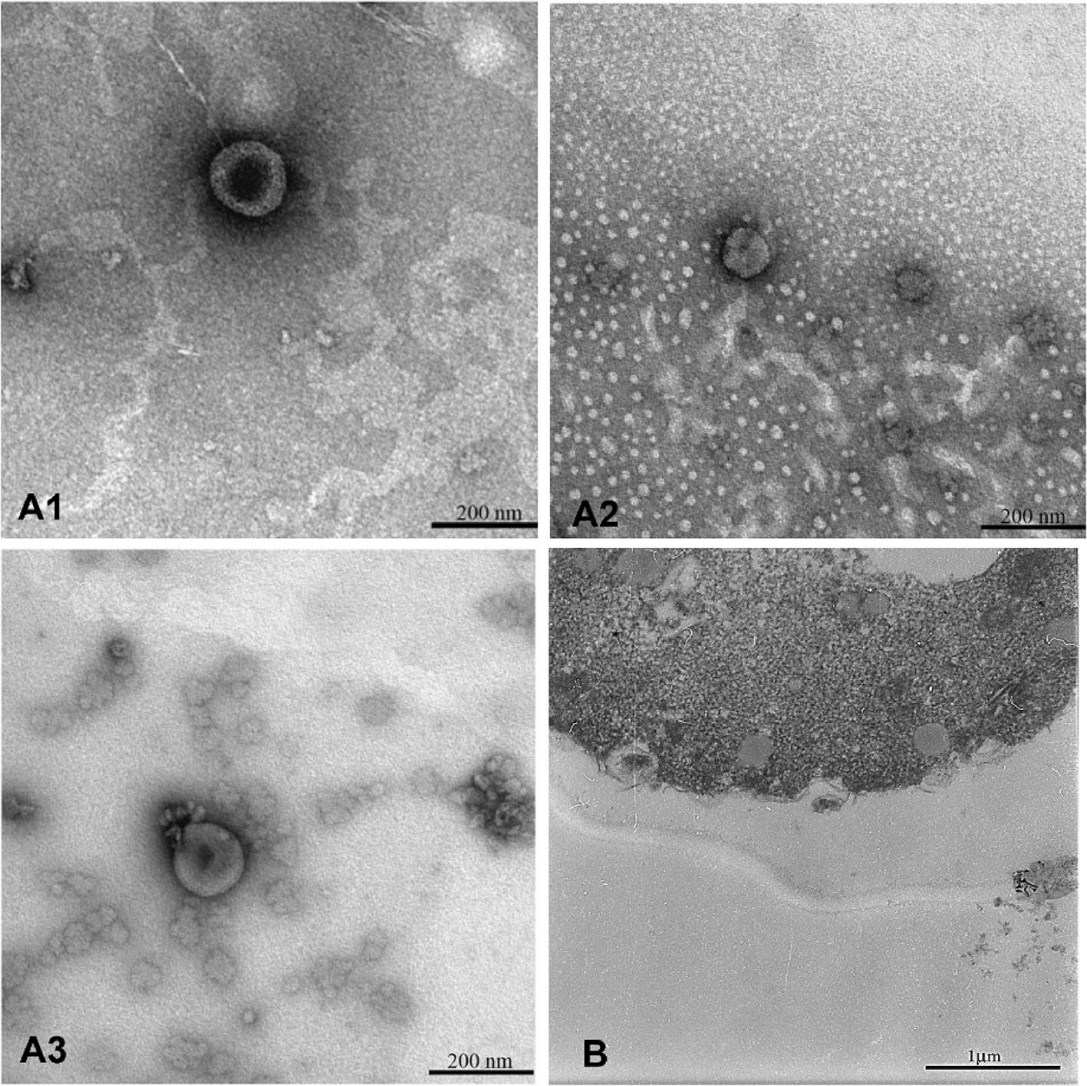
Transmission Electron Microscopy observations of yeast EVs. The scale bars are indicated in each picture. (**A1**–**A3**) TEM images of EVs isolated from the three yeast strains, CL4 (Sc), CL1(Pf), and ts9 (Sc), respectively. (**B**) TEM images of a *Pichia fermentans* cell releasing vesicles-like structures.

TEM analyses (Fig. 2) showed the typical cup-shaped forms and dimensions of yeast extracellular vesicles (2.A1-3), in accordance with those present in the literature^5,7^. We were able to capture images of sections of yeast cells with vesicular bodies crossing the cell wall (2.B).

### Yeast EVs impact the immunogenicity of human DCs

**Figure 3:**
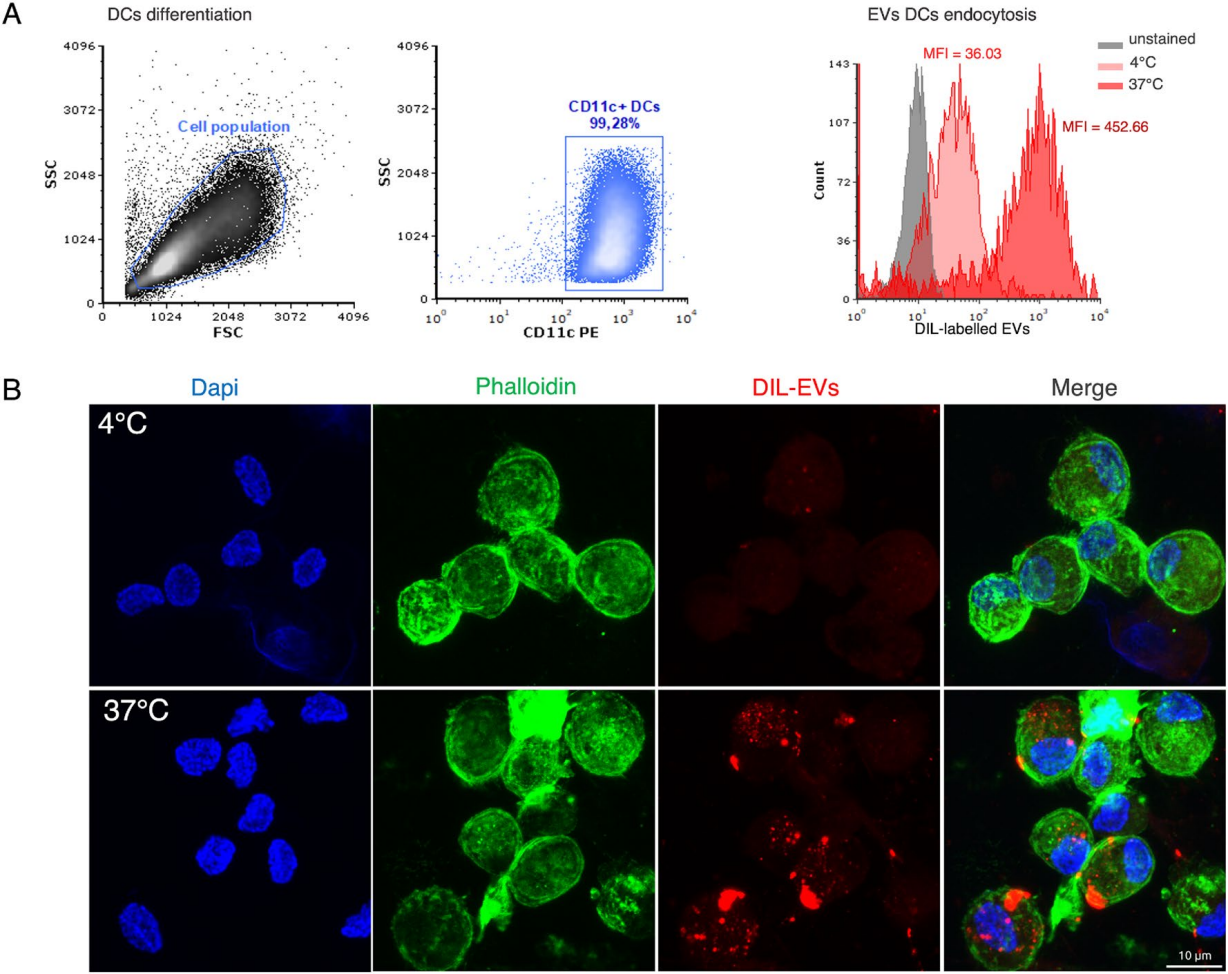
Flow cytometry and confocal microscopy evaluation of EVs endocytosis by DCs. (**A**) Flow cytometry gating strategy used to detect monocyte-derived mature (CD11c +) DCs after 7 days of differentiation. DCs are included in the “Cell population” gate (upper left dot plot), on the total of captured events, in SSC (side scatter) versus FSC (forward scatter) plot. The upper right dot plot shows the percentage of CD11c + cells versus SSC within the selected cell population. The lower plot reports the histograms of DiI-labelled fluorescent EVs within the DCs versus events count at 4 °C (light red histogram) or 37 °C (dark red histogram); the gray histogram reports the auto-fluorescence of unstained control cells. Fluorescence was expressed as Mean Fluorescence Intensity (MFI). Events number of histogram overlays was normalized based on the maximum peak value. (**B**) EVs endocytosis by DCs captured by confocal microscopy after DCs-EVs incubation at 4 °C (upper row) or at 37 °C (lower row). DCs nuclei and cytoskeleton F-actin were stained with DAPI (in blue, first column) and phalloidin (in green, second column), respectively; EVs were labeled by DiI dye (in red, third column). Merge is shown in the fourth column. Scale bar = 10 μm. Image size: 50 × 50 μM.

We first evaluated EVs endocytosis by Mo-DCs by flow cytometry and confocal microscopy. The endocytosis assay was performed on 7-day differentiated CD11c + Mo-DCs (Fig. 3A, left dot plots). Flow cytometry analysis showed that Mo-DCs effectively internalized EVs at 37 °C (MFI = 452.66), while reduced internalization was observed at 4 °C (MFI = 36.03), used as endocytosis negative control (Fig. 3A, right histogram plot). To further confirm the EVs uptake by Mo-DCs and visualize DiI-stained EVs with respect to cell cytoskeleton F-actin distribution and nuclei, samples were investigated by confocal microscopy (Fig. 3B). Endocytosed DiI-EVs (in red) are visible and mainly distributed within Mo-DCs cytoplasm, with an appreciable difference between 37 °C and 4 °C, consistent with the flow cytometry observation.

**Figure 4:**
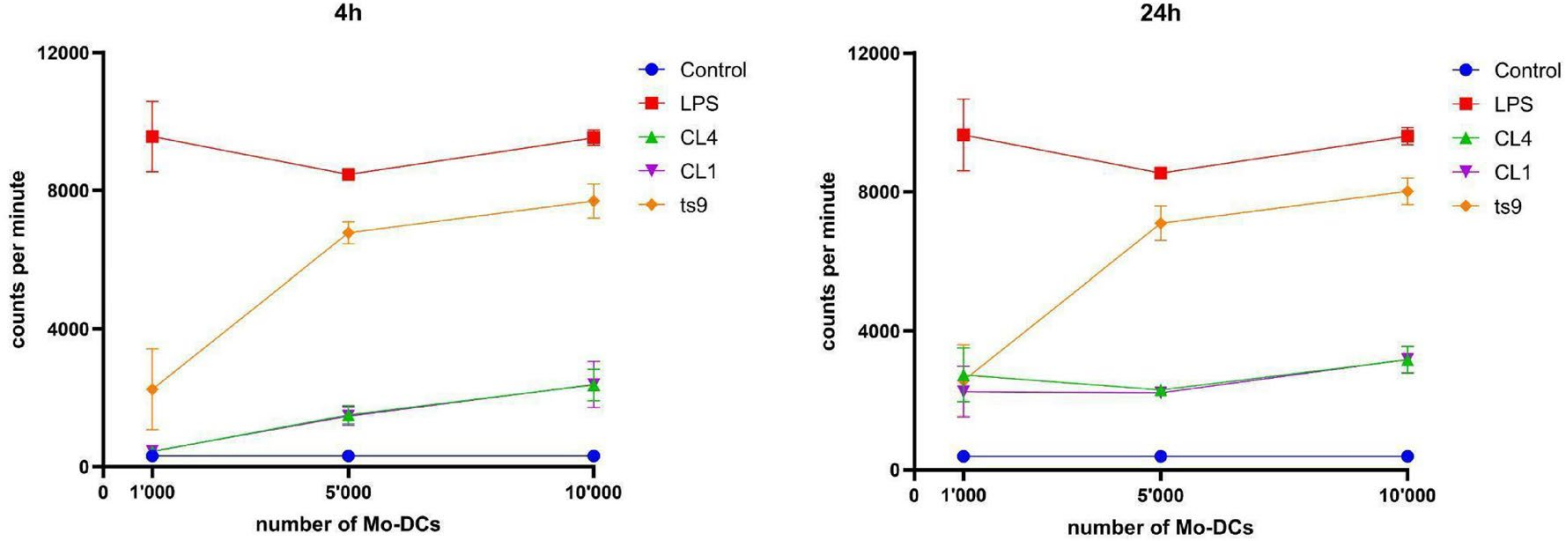
Evaluation of proliferation levels of CD4 + T cells through a mixed lymphocyte reaction. Graphs report the results of MLR as count per minute, evaluated by ^3^H-thymidine incorporation assay (see ‘Methods’ section), after 4 h (left graph) or 24 h (right graph). Allogeneic T cells were plated alone as control (blue line) or with Mo-DCs stimulated with LPS (red line) or after the incubation with CL4 (Sc) (green line), CL1 (Pf) (violet line), or ts9 (Sc) (orange line). Means and standard errors for four independent experiments are reported. Statistics and graphs were generated using GraphPad Prism 6 software. Raw data are shown in Supplementary Material (Table S6).

To assess Mo-DCs activation and function after interaction with yeast EVs, we performed a mixed lymphocyte reaction (MLR) with lipopolysaccharide (LPS) activated Mo-DCs and allogeneic T cells. The incubation of LPS-activated Mo-DCs with yeast EVs (Fig. 4) significantly reduced the proliferation of allogeneic T cells compared to the control group (LPS-activated Mo-DCs without EVs), both after 4 or 24 h of incubation (*p* values < 0.05, Figure S1). CL4 (Sc) and CL1 (Pf) samples (EVs deriving from the fermented milk strains) induced a stronger impairment in Mo-DCs functionality than the laboratory strain ts9 (Sc). This effect was more consistent as Mo-DCs concentrations increased (5 × 10^3^ and 10^4^), suggesting a dose-dependency, as shown in Supplementary material (Figure S1). Moreover, the 1:20 dilution was slightly more effective on average than the 1:200 one (Figure S1).

### Small RNA content changes according to the yeast isolation source

**Figure 5:**
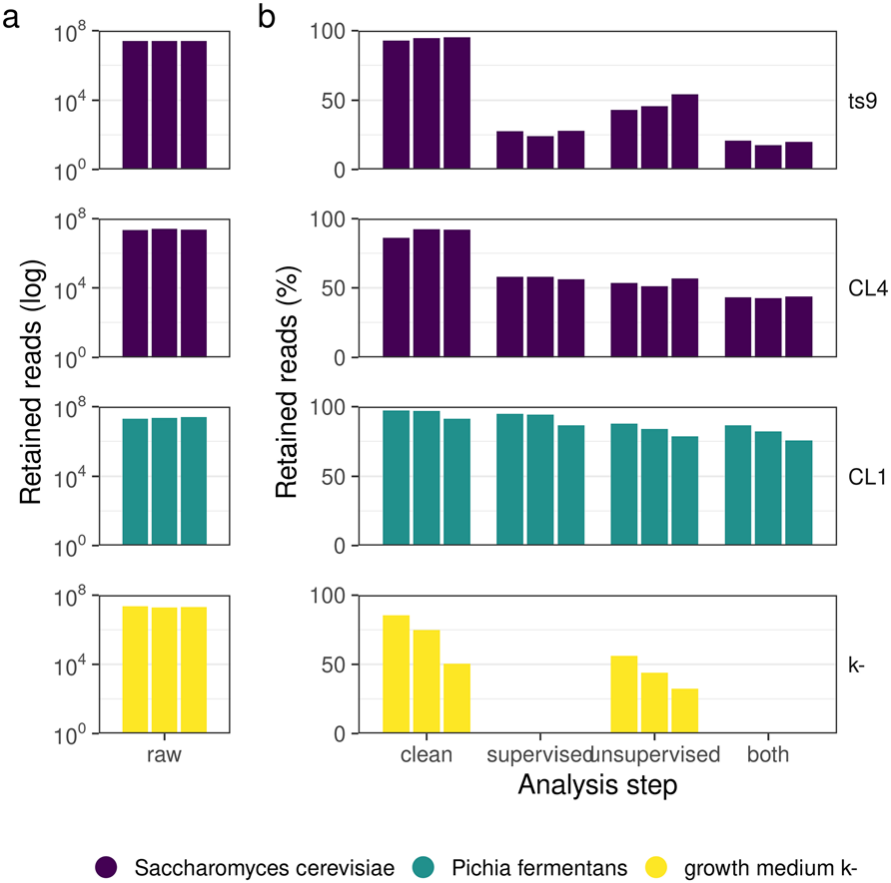
Number of sequences retained after identification and removal of ribosomal sequences. The number of raw reads per sample is reported in the “**a**” panel whereas the percentage of reads retained after filtering out putative ribosomal sequences is reported in “**b**”. The analysis steps are reported in the x-axis: raw, number of raw reads sequenced; clean, percentage of reads retained after quality filtering; supervised, percentage of reads retained after aligning reads to a custom ribosomal dataset; unsupervised, percentage of reads retained after Ribodetector pipeline; both, percentage of reads retained after both supervised and unsupervised approaches. The analyzed samples were K- (yeast growth medium) and EVs produced by the strains CL4 (*S. cerevisiae* from fermented milk), CL1 (*P. fermentans* from fermented milk) and ts9 (*S. cerevisiae* from laboratory collection).

Following RNAse treatment of EVs to exclude contamination from carryover of cellular RNAs, small RNA fractions (< 200 nt) were extracted from 3 replicates of EVs from the 3 yeast strains and then sequenced. Quality Control (QC) analyses confirmed the presence of small RNA peaks and high amounts of RNA (Supplementary material, Figure S2). More than 75% of raw sequences from both *Saccharomyces cerevisiae-* (CL4 and ts9) and *Pichia fermentans-*derived EVs (CL1) were retained after the quality filtering step, whereas K- samples (Negative Control, see Material and Methods section) reported lower values. The ribosomal sequence analysis resulted in the removal of around 50% of the ts9 and CL4 reads (*Sc*), while more than 75% of the CL1 (*Pf*) sequences were retained (Fig. 5).

**Figure 6:**
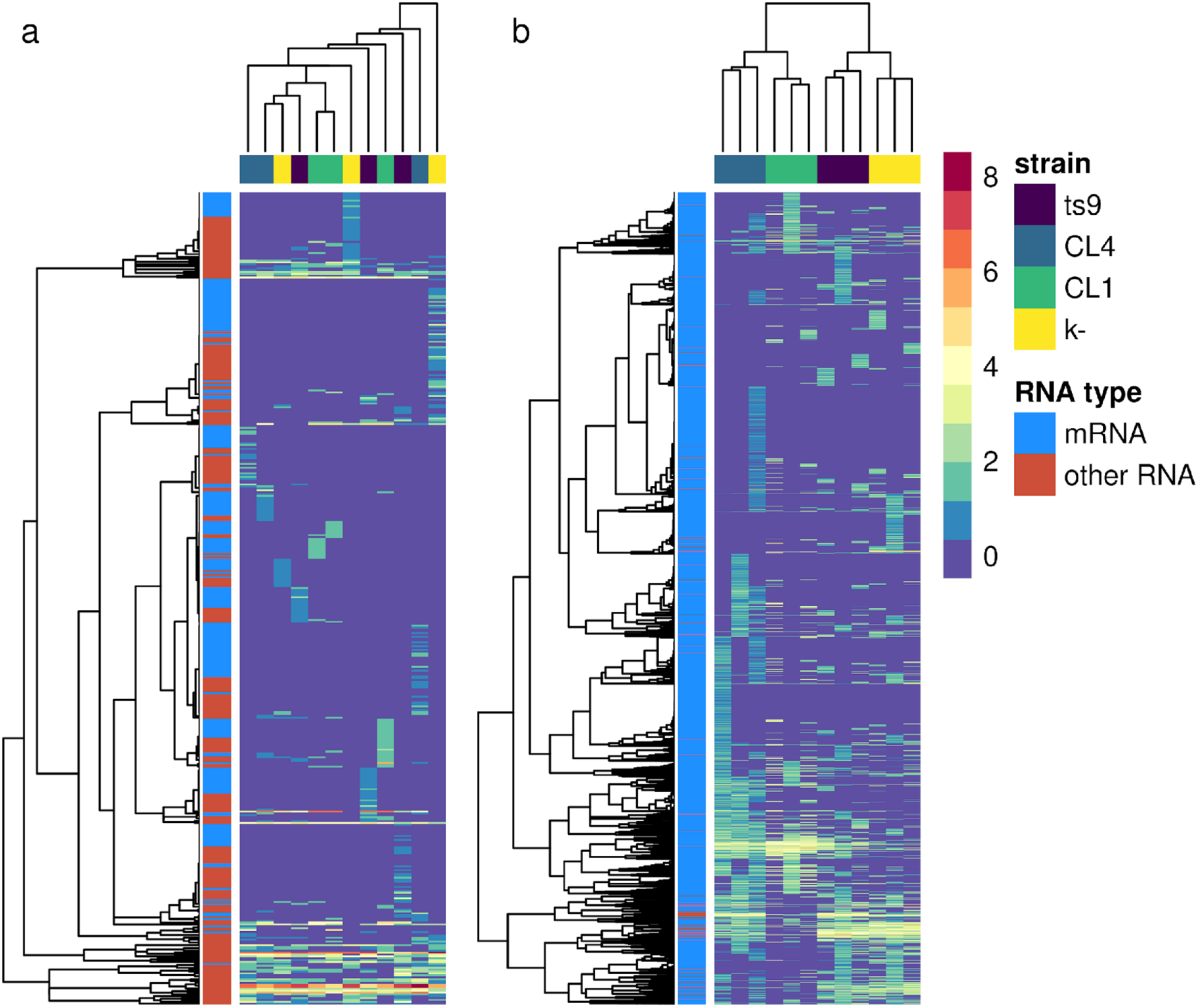
RNA mapping reads against human and yeast transcriptomes. Scaled abundance of genes detected by Salmon (a tool for quantifying the expression of transcripts using RNA-seq data). Transcript counts were transformed by applying the variance stabilizing transformation (VST) implemented in DESeq2. Transformed values were then centered and scaled to be better compared. Transcript and sample dendrograms were reported in the top and left parts of the diagrams, respectively. Yeast strains producing the EVs were reported in the top part of the heatmap, whereas gene types were reported on the left. The two panels report the scaled abundance obtained by mapping RNA reads against the “**a**”, human and “**b**” yeast transcriptome. The analyzed samples were K- (yeast growth medium) and EVs produced by the strains CL4 (*S. cerevisiae* from fermented milk), CL1 (*P. fermentans* from fermented milk), and ts9 (*S. cerevisiae* from laboratory collection). Statistics and graphs were generated using R software (v4.2).

EVs were shown to contain mainly fragments of fungal mRNA, with a small percentage of non-coding sequences (Figure S5). Mapping of these sequences against the human genome also resulted in a relative majority of human mRNA sequences, but the analysis of consistency between EVs replicates revealed that most of the sequences that mapped on human mRNA were present only in one replicate (Figure S6). A summary table of retained reads after each analysis step (quality filtering, ribosomal sequence removal, and human and *S. cerevisiae* mapping) is provided in Supplementary material (Table S1). Gene abundance on the *S. cerevisiae* reference reported strong clustering according to different strains and controls, whereas no clustering resulted from the analysis of the human genome (Fig. 6).

**Figure 7:**
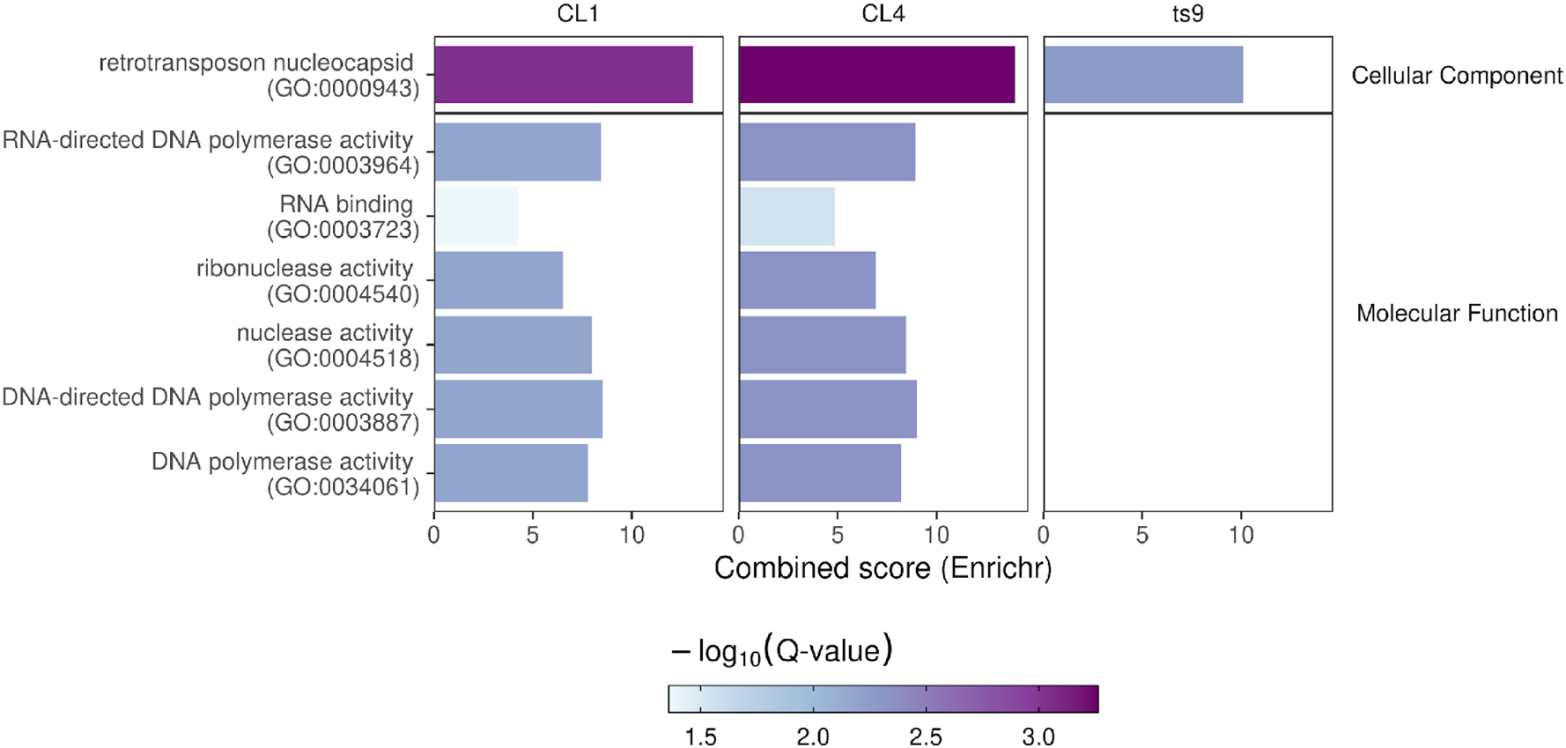
Enrichment analysis of differentially abundant genes against control. Yeast strains were reported in the top part of each panel whereas GO categories significantly enriched were reported in the y-axis and divided according to broad GO categories. Transcript enrichment was assessed by using the modified Fisher test implemented in Enrichr. All significant categories were reported together with their “combined score” (x-axis) which accounts for both *p* value and Z-score. Bars were colored according to the adjusted *p* value (*Q* value). Statistics and graphs were generated using R software (v4.2).

Enrichment analysis of differentially abundant genes (Fig. 7) showed that EVs of the three yeast strains were enriched for the retrotransposon nucleocapsid cellular component, whereas the other six molecular functions are shared only by *Saccharomyces cerevisiae* CL4 and *Pichia fermentans* CL1.

The enriched cellular component GO:0,000,943 is associated with the retrotransposon nucleocapsid encoded by genes of the Ty class, indicating the enrichment of transposable sequences in the vesicles. Furthermore, the function 00,003,964 encodes an RNA-based DNA polymerase with reverse transcriptase activity, and other DNA polymerase genes are present only in the fermented milk isolated strains but not in the *S. cerevisiae* ts9 strain.

## Discussion

Fungal extracellular vesicles are being studied by the scientific community as mediators of the immune response^5,7^. They are recognized as pathogen-associated molecular patterns (PAMPs) by the pattern recognition receptors (PRRs) of the host's innate immune system. Therefore, fungal EVs could modulate the activation of the immune response^43^ which may depend either on the fungal species or the EVs structure and content^44^. The studies on the immunomodulatory properties of fungal EVs suggest that they can play an important role in host-fungal communication, exerting a plethora of different effects that range from the enhancement of the host immune response to a control of the fungal infection or tolerance by the immune system^45–48^.

To date, studies on EVs have mainly focused on pathogen species and infection dynamics, although there is currently a scientific consensus on the fact that also foodborne, transient, and stable commensal species can modulate the host immune response^49,50^. Some yeast species, such as *Saccharomyces cerevisiae*, *Pichia fermentans,* and *Kluyveromyces marxianus*, are associated with fermented foods known to have probiotic properties^51^. To date, the mechanisms behind the beneficial effects provided by foodborne microorganisms have only been partially established. There is a growing scientific consensus on the hypothesis that the probiotic properties of functional foods could be mediated not only by the metabolic products of the fermentative community, such as Short-Chain Fatty Acids (SCFAs) but also through the release of EVs^52,53^. Probiotic yeast EVs have been found not to be detrimental to human gastrointestinal health^54^. To test the hypothesis of a potential beneficial role of yeast EVs, we investigated the immunomodulatory properties of EVs from yeasts isolated from fermented goat milk (Yaghnob Valley, Tajikistan)^29^ and compared them to EVs produced by a wine *S. cerevisiae* strain. Results showed that vesicles produced by the three yeast strains have comparable physical characteristics, as commonly found in the literature^55^. We then confirmed the uptake of EVs by human dendritic cells through flow cytometry assays and confocal microscopy. The reduced internalization at 4 °C compared to 37 °C (about 12-fold diminished) suggests that the EVs uptake by Mo-DCs may be an active mechanism such as an endocytosis. Then, we observed that only EVs from the fermented milk strains, i.e. CL4 (Sc) and CL1 (Pf), are able to significantly reduce the antigen-presenting ability of DCs, previously activated with an LPS stimulus. Our results allow us to speculate that one of the mechanisms underlying the beneficial effects of fermented beverages on human health could derive from the release of specific yeast EVs and their subsequent interactions with the host immune system. Moreover, since vesicles of the *S. cerevisiae* strain (ts9) from a different source (wine grapes) impact the activation of DCs to a much lesser extent, we hypothesize that the anti-inflammatory properties of yeast EVs are not species-specific, but strain-specific, and that depend on the different evolutionary trajectories of strains from different ecological niches. In fact, it is known that wine strains, fermented food strains, and human-derived strains have different genetic makeup and immunomodulatory properties, whose determinants can only in part be attributed to cell wall characteristics^50^. Our findings propose specific yeast strains as a source of EVs of interest for the delivery of anti-inflammatory molecules in clinical applications.

The delivery of regulatory RNA (miRNA-like molecules, siRNAs, t-RNA fragments) carried by EVs into target cells is a known communication mechanism between the microbe and host^17,18,23^. In fungi, several studies prove that RNA interference is mediated by fungal EVs in models of plant infection^22,56^ and in a study on mosquitoes^23^. However, as far as we know, there is no evidence of fungal EVs that show RNA interference in human cells^57^. In our study, we sought to find a possible RNA-mediated mechanism exerted by the EVs cargo, underlying the observed strong anti-inflammatory effect on human DCs. Sequencing of the small RNA fraction (enriched in molecules shorter than 50 nt) internalized by EVs of these yeast strains identified diverse non-coding RNAs, in accordance with previous findings^18,19^. However, most of the RNA sequences found in the studied fungal EVs were segments of mRNA transcripts, possibly suggesting a role for EVs in the excretion of degraded mRNAs. We hypothesize that some of the mRNA fragments found in EVs could be the result of RNA turnover in the yeast cells, where the regulation based on the stability of the transcript is a key biological process. Out of the 6275 putative genes in the *S. cerevisiae* genome^58^, we found that mRNA fragments in EVs mapped to approximately 4000 *S. cerevisiae* genes, suggesting that a large part of the strain’s degraded RNA pool was carried by these vesicles. Mapping the reads against the human genome showed around 200 target mRNA transcripts, but without consistency among the EVs triplicates, suggesting the unlikelihood of an association of the immune modulatory effect observed for Yeast EVs with specific RNA interference of yeast sRNAs with human transcripts. Of particular interest was the discovery that sRNAs from the cellular component “retrotransposon nucleocapsid” (GO:0,000,943) were enriched in all three strains tested. This component contains RNAs matching the long-terminal repeat (LTR) retrotransposons genes of the Ty class. The genome of *Saccharomyces cerevisiae* S288C (the reference strain) harbors 5 types of LTR retrotransposon, from Ty1 to Ty5, that duplicate themselves by reverse transcription of their RNA genome. Ty genes are considered the evolutionary ancestors of retroviruses^59^ and the S288c *S. cerevisiae* genome shows recent insertions in Ty genes from related species through horizontal gene transfer^59,60^. It is also known that Tys can be used to trace the origin and evolutionary history of yeast strains^61^. The number of Ty1 elements in the genomes of different strains varies significantly, also depending on the source and the geography^62^. Little is known about how yeasts exchange Tys besides rare mating events. It is possible that strains with more Tys contain vesicles with an enrichment in Tys levels, and our results could be the first evidence that vesicles act as vectors of Tys in horizontal gene transfer, supporting the importance of this mechanism in Ty transmission between strains^60^.

It is noteworthy that other sRNAs mapping to ORFs with molecular functions related to RNA-based DNA polymerase with reverse transcriptase activity (function 00,003,964), and other RNA binding, ribonuclease activity, and polymerase activities (Fig. 7) were enriched only in the fermented milk strains but not in the vineyard *S. cerevisiae*. The enrichment of these specific RNAs in the fungal EVs of strains CL4 (Sc) and CL1 (Pf) but not in ts9 (Sc) could be associated with the different strain-specific anti-inflammatory effects on human DCs, providing a testable working hypothesis for further investigations. The absence of targets for RNAi can suggest possible alternative immune modulation processes exerted by the RNA content of EVs on DCs, involving the binding of small RNAs with toll-like receptors (TLR) and impairment of immune signaling, dampening inflammation, as previously explored by our work^63^. As well as further investigation on other possible regulatory activity exerted by RNAs, an important step to explore the biological functions of yeast EVs would be the caracterization of the protein cargo through a proteomic analysis.

In conclusion, our results provide insights into the potential clinical applications of non-pathogenic yeast EVs as suitable candidates for the delivery of immunomodulatory molecules in several human conditions.

### Supplementary Information

Supplementary Information 1.

Supplementary Information 2.

Supplementary Information 3.

Supplementary Information 4.

## Data Availability

Sequences were deposited into the “European Nucleotide Archive'' (ENA) under the accession: PRJEB59224.
