# Supplementary material for: Yeast strains isolated from fermented beverage produce extracellular vesicles with anti-inflammatory effects
Source: Sci Rep. 2024 Jan 6;14:730. doi: 10.1038/s41598-024-51370-7 (PMC10771474; doi:10.1038/s41598-024-51370-7)
Supplement: Supplementary file 1 — Supplementary Information 1. [file 41598_2024_51370_MOESM1_ESM.pdf]

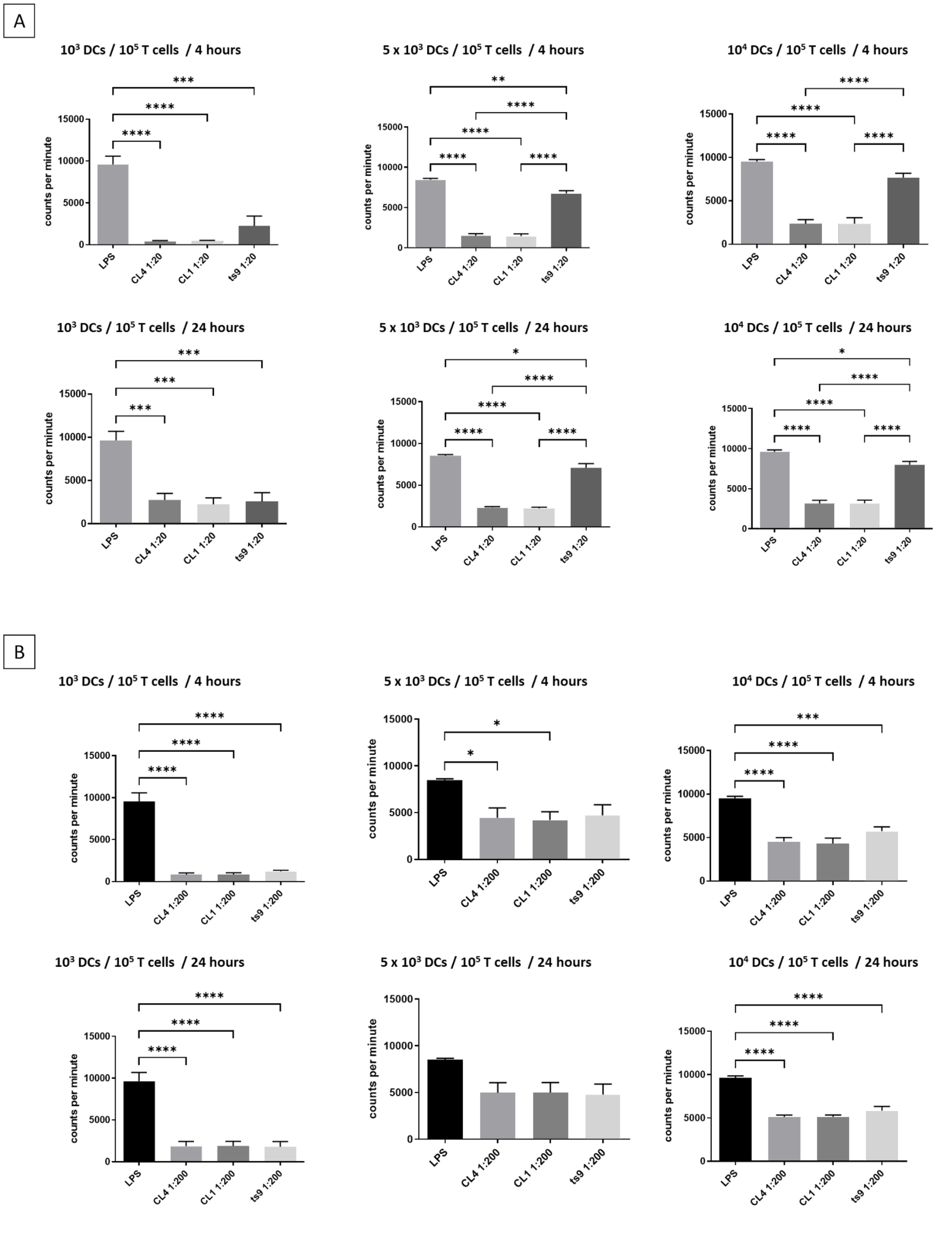


**Figure S1.** Evaluation of proliferation levels of CD4+ T cells through Mixed Lymphocyte Reaction. Allogenic T cells have been stimulated with 3 different concentrations of dendritic cells previously incubated with 2 different dilutions of yeast EVs (A = dil 1:20, B = dil 1:200) for 4 or 24 hours. The T

cells proliferation has been evaluated as counts per million of incorporated tritiated thymidine with a Microbeta 1450 Trimux counter (Wallac). Graphs show means and standard errors for 3 independent experiments made with distinct EVs preparations. Statistics and graphs were generated using GraphPad Prism 6 software.

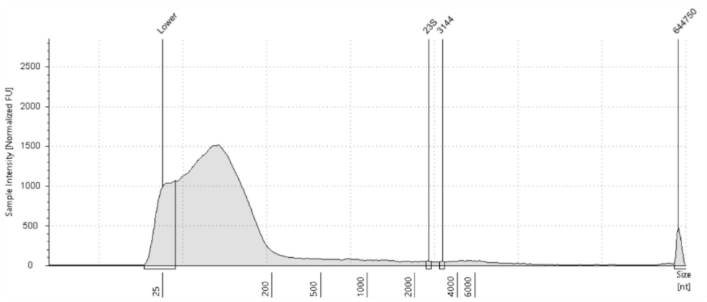


| Sample ID | CL4 |
|---|---|
| Volume (µl) | 25 |
| Concentration (ng/µl) | 28.1 |
| Total Amount (ng) | 702.5 |

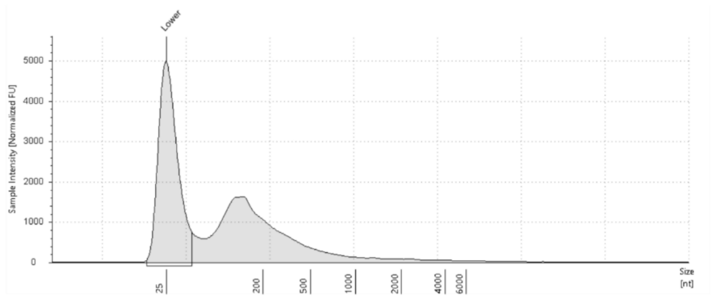


| Sample ID | CL1 |
|---|---|
| Volume (µl) | 25 |
| Concentration (ng/µl) | 35.1 |
| Total Amount (ng) | 877.5 |

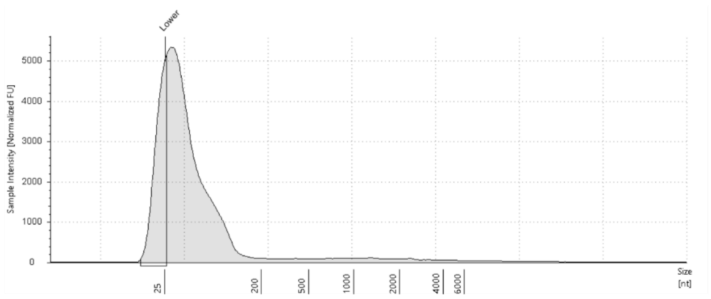


| Sample ID | ts9 |
|---|---|
| Volume (µl) | 25 |
| Concentration (ng/µl) | 87.4 |
| Total Amount (ng) | 2145.3 |

**Figure S2.** Summary of small RNA samples' QC analyses with RNA tape station (Agilent Technologies, Santa Clara, California, U.S.) on the left and quantification with Qubit (Thermo Fisher Scientific, Waltham, Massachusetts, U.S.) on the right.

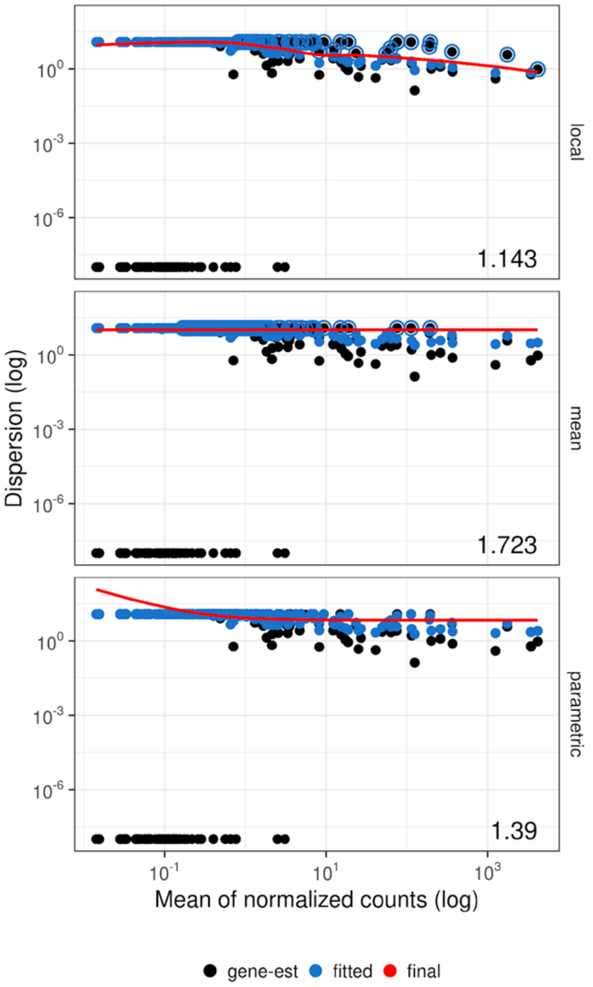


**Figure S3.** Gene dispersion models evaluation in the human transcriptome. Different model types were tested before fitting gene dispersion with DESeq2. The model type was reported in the right side of each panel whereas gene dispersion and mean abundance were reported in the y and x-axes, respectively. The median absolute residual value was reported in the bottom right corner of each panel. Black points represent the estimated gene dispersion whereas blue ones represent the dispersion after model fitting. Red lines represent final models fitted by DESeq2. Statistics and graphs were generated using R software (v4.2).

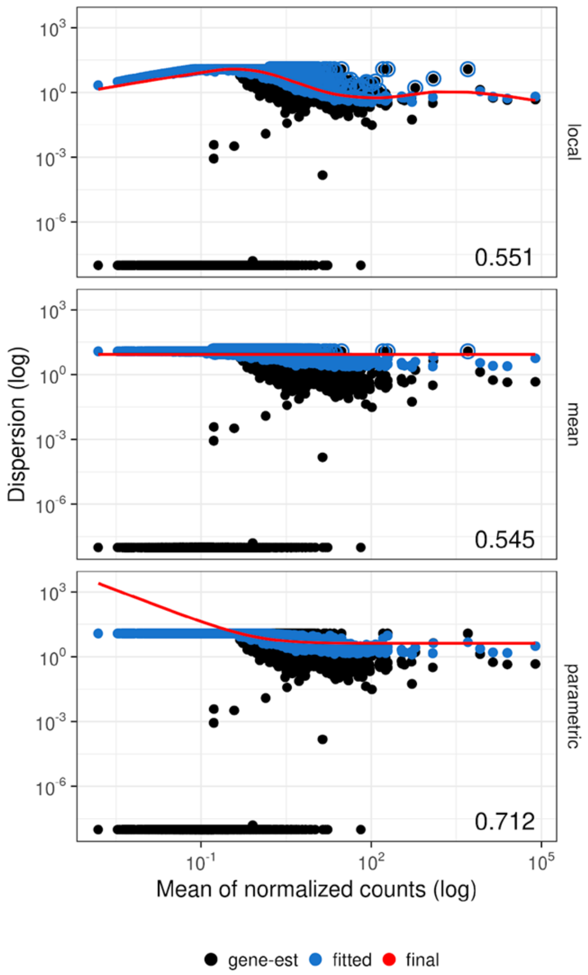


**Figure S4.** Gene dispersion models evaluation in the *Saccharomyces cerevisiae* transcriptome. Different model types were tested before fitting gene dispersion with DESeq2. The model type was reported in the right side of each panel whereas gene dispersion and mean abundance were reported in the y and x-axes, respectively. The median absolute residual value was reported in the bottom right corner of each panel. Black points represent the estimated gene dispersion whereas blue ones represent the dispersion after model fitting. Red lines represent final models fitted by DESeq2. Statistics and graphs were generated using R software (v4.2).

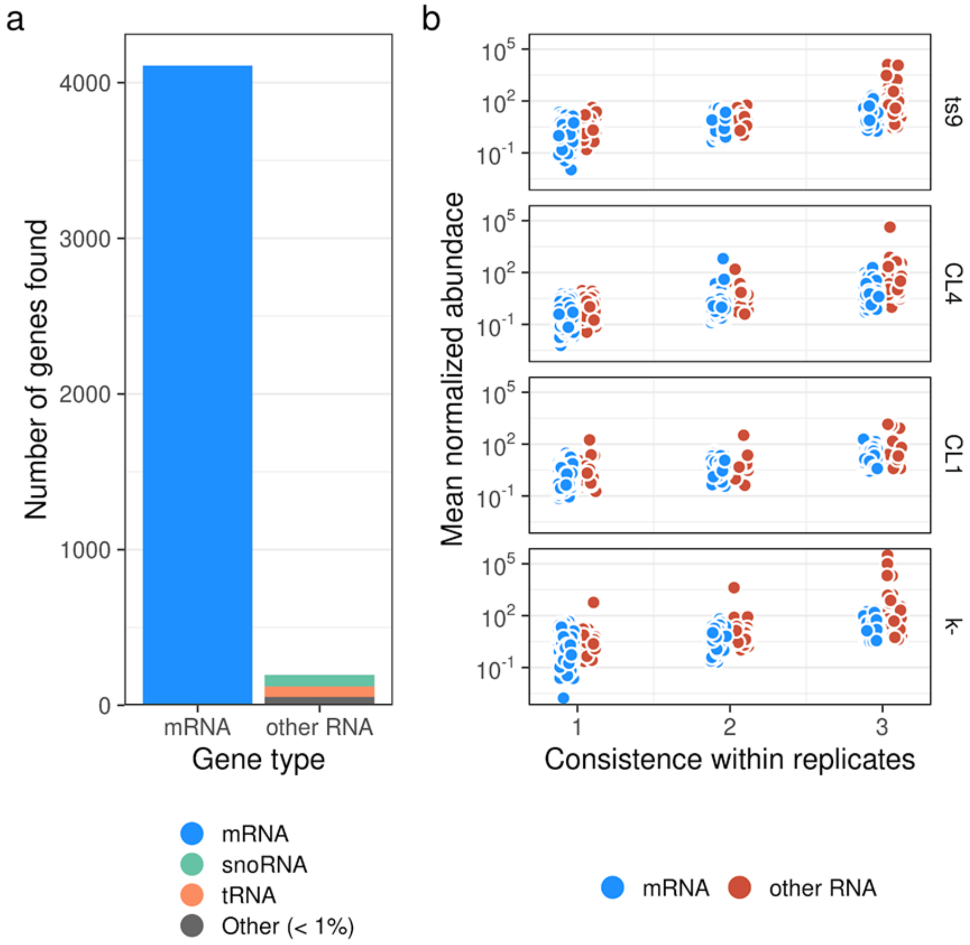


**Figure S5.** Types of genes detected in the *Saccharomyces cerevisiae* transcriptome. The transcript type was reported in panel "a" whereas their consistency within replicates and their mean abundance value was reported in panel "b". Gene types were divided into coding (mRNA) and not-coding (other RNA) in both panels. Statistics and graphs were generated using R software (v4.2).

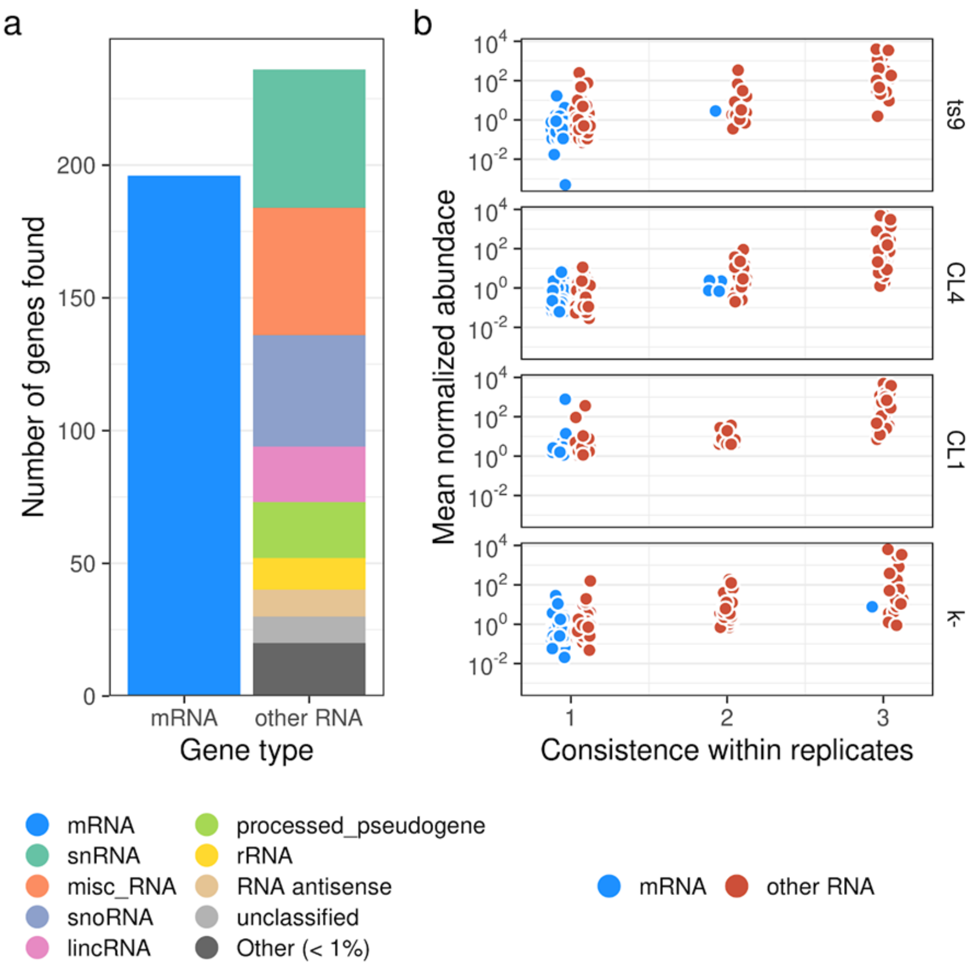


**Figure S6.** Types of transcripts detected in the human transcriptome. The number of different transcripts was reported in panel "a" together with their type, whereas the consistency within replicates and the mean abundance values were reported in panel "b". Transcript types were divided into coding (mRNA) and not-coding (other RNA) in both panels. Statistics and graphs were generated using R software (v4.2).

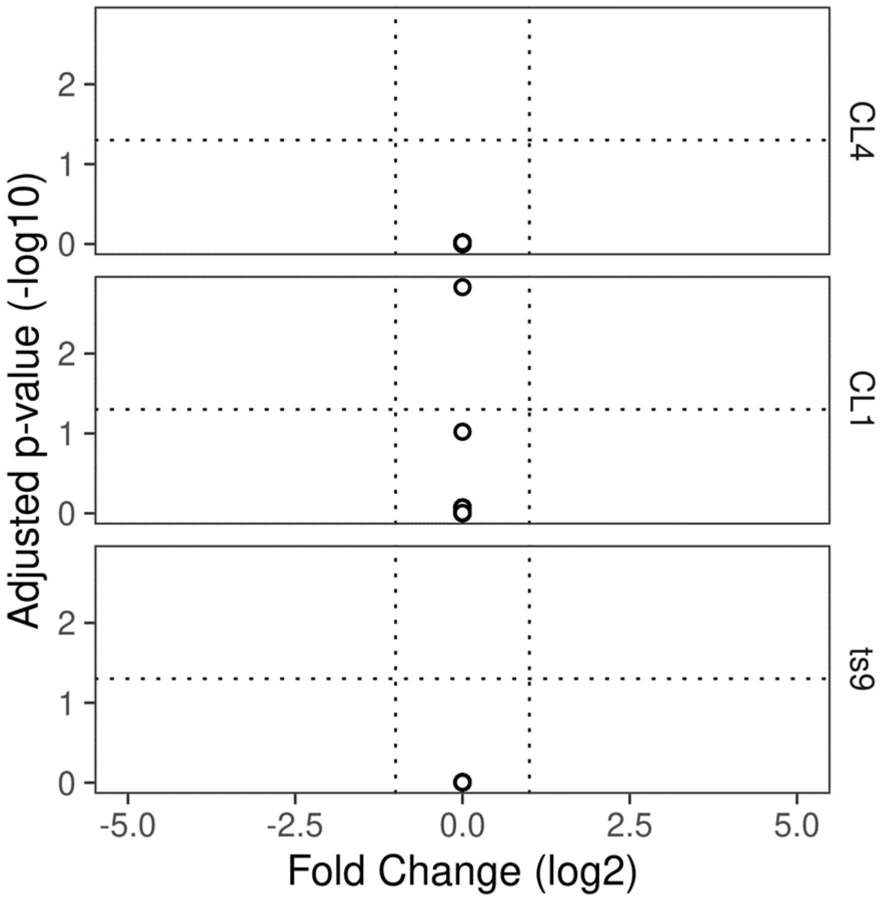


**Figure S7.** Differential abundance analysis results (human transcriptome). Fold change values and p-values were reported in the y and x-axis, respectively. Dotted grey lines represent thresholds used for selecting significant transcripts ($p < 0.05$ and fold change $> 1$).

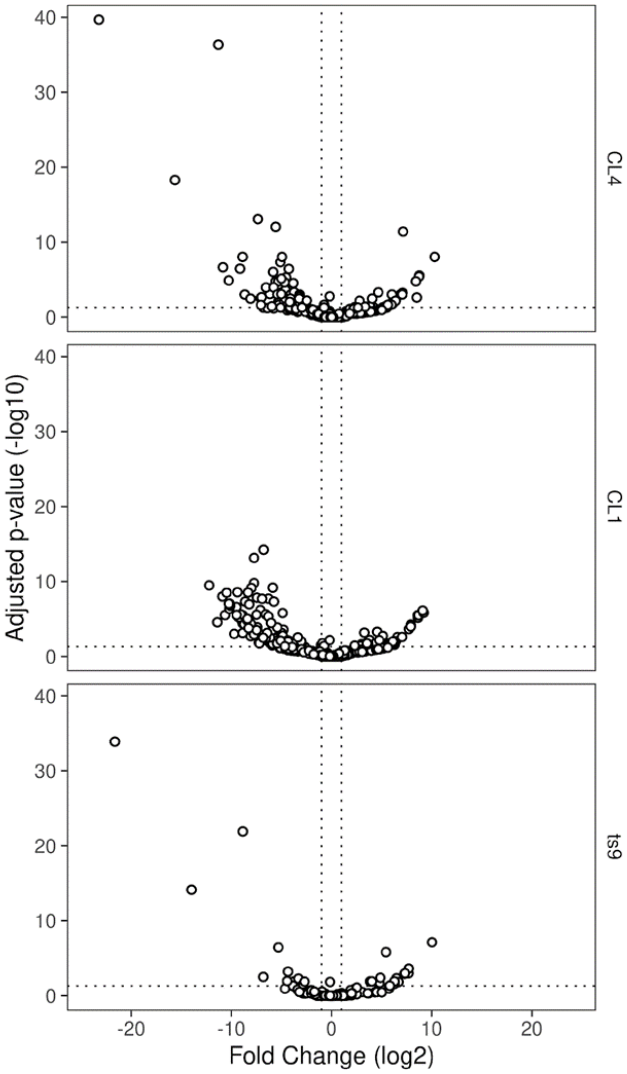


**Figure S8.** Differential abundance analysis results (yeast transcriptome). Fold change values and p-values were reported in the y and x-axis, respectively. Dotted grey lines represent thresholds used for selecting significant transcripts ($p < 0.05$ and fold change > 1).

**Table S1.** Number of reads retained after each analysis step. The number of reads obtained after filtering out putative ribosomal sequences was reported in the "Filtered Reads" column together with the number of reads that was mapped against the human transcriptome ("Human Mapped") and the *Saccharomyces cerevisiae* transcriptome ("Yeast Mapped").

| Species | Filtered Reads | Human Mapped | Yeast Mapped |
|---|---|---|---|
| Saccharomyces cerevisiae ts9 | 5335567 | 72383 | 32851 |
| Saccharomyces cerevisiae ts9 | 5150050 | 4692 | 13020 |
| Saccharomyces cerevisiae ts9 | 4513077 | 13830 | 44456 |
| Saccharomyces cerevisiae CL4 | 11018189 | 164238 | 208835 |
| Saccharomyces cerevisiae CL4 | 10364937 | 19696 | 37644 |
| Saccharomyces cerevisiae CL4 | 9400118 | 33364 | 78723 |
| Pichia fermentans CL1 | 18772397 | 2943 | 9391 |
| Pichia fermentans CL1 | 17194009 | 2874 | 3193 |
| Pichia fermentans CL1 | 18339699 | 2423 | 5641 |
| growth medium k- | 8638760 | 38468 | 614403 |
| growth medium k- | 6452447 | 14398 | 185366 |
| growth medium k- | 12743058 | 9939 | 297327 |

**Table S2 (separate file).** Results of differential abundance analysis performed with DESeq2 on the human transcriptome. Yeast strains tested were reported in the “Contrast” column whereas the transcript ID (according to the Ensembl database) was reported in the “Ensembl ID” column. The overall mean abundance of each transcript (normalised according to DESeq2) was reported in the “Mean Abundance” column whereas the logarithm in base two of the fold-changes relative to each contrast were reported in the “Fold-change (log2)” column. The P-value and the adjusted P-value (Benjamini-Hochberg correction) were reported in the “P-value” and “Q-value” columns, respectively.

**Table S3 (separate file).** Results of differential abundance analysis performed with DESeq2 on the *Saccharomyces cerevisiae* transcriptome. Yeast strains tested were reported in the “Contrast” column whereas the transcript ID (according to the Ensembl database) was reported in the “Ensembl ID” column. The overall mean abundance of each transcript (normalised according to DESeq2) was reported in the “Mean Abundance” column whereas the logarithm in base two of the fold-changes relative to each contrast were reported in the “Fold-change (log2)” column. The P-value and the

adjusted P-value (Benjamini-Hochberg correction) were reported in the “P-value” and “Q-value” columns, respectively.

**Library construction, quality control and sequencing.** Briefly, 3’ and 5’ adaptors were ligated to 3’ and 5’ end of small RNA, respectively. Then the first strand cDNA was synthesized after hybridisation with reverse transcription primer. The double-stranded cDNA library was generated through PCR enrichment. After purification and size selection, libraries with insertions between 18~40 bp were ready for sequencing with SE50. The library was checked with Qubit and real-time PCR for quantification and bioanalyzer for size distribution detection. Quantified libraries were then pooled and sequenced on Illumina platforms, according to effective library concentration and data amount required.

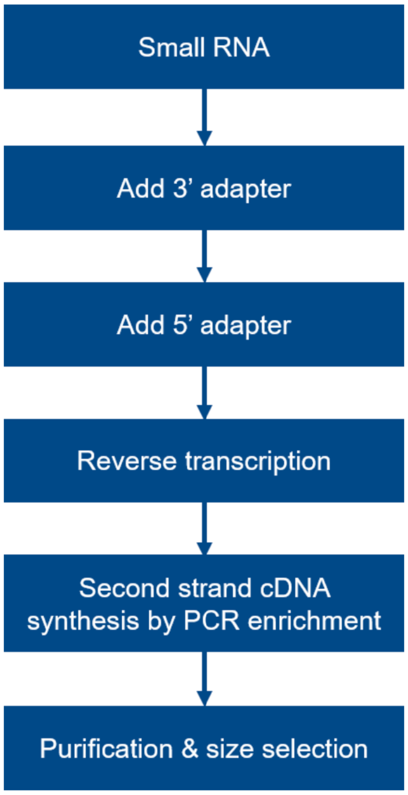


**Table S4.** Summary of data quality analysis. Q20(%) and Q30(%) are the percentages of bases on the total base number that have a Phred value of more than 20 or 30, respectively. GC is the number

of G and C bases on the total base number.

| Sample | Raw reads | Raw data(G) | Error(%) | Q20(%) | Q30(%) | GC(%) |
|---|---|---|---|---|---|---|
| A1 | 21790669 | 1.1 | 0.01 | 99.16 | 97.24 | 53.02 |
| A2 | 25886953 | 1.3 | 0.01 | 99.07 | 97.22 | 53.08 |
| A3 | 23641594 | 1.2 | 0.01 | 99.29 | 97.69 | 53.32 |
| B1 | 19895748 | 1.0 | 0.01 | 99.47 | 98.03 | 49.55 |
| B2 | 22853374 | 1.1 | 0.01 | 99.47 | 98.01 | 50.28 |
| B3 | 24302422 | 1.2 | 0.01 | 99.42 | 97.78 | 50.62 |
| C1 | 25483670 | 1.3 | 0.01 | 99.28 | 97.48 | 56.15 |
| C2 | 25579372 | 1.3 | 0.01 | 99.32 | 97.52 | 55.69 |
| C3 | 25790991 | 1.3 | 0.01 | 99.39 | 97.87 | 55.24 |
| D1 | 22694237 | 1.1 | 0.01 | 99.10 | 97.05 | 53.46 |
| D2 | 19709676 | 1.0 | 0.01 | 98.77 | 96.43 | 53.25 |
| D3 | 19975378 | 1.0 | 0.01 | 97.99 | 95.13 | 51.75 |

**Data filtration.** To get the final clean reads, the unpurified raw reads was processed as follows:

(1) Get rid of reads of which more than 50% bases has a base quality score no more than 5;

(2) Get rid of reads containing N > 10%;

(3) Get rid of reads with 5' primer contaminants;

(4) Get rid of reads without 3' primer and reads without the insert tag;

(5) Trim 3' primer sequence;

(6) Get rid of reads with polyA/T/G/C.

Small RNA adapter sequences:

RNA 5’ Adapter (RA5), part: 5'-GTTCAGAGTTCTACAGTCCGACGATC-3'

RNA 3' Adapter (RA3), part: 5'-AGATCGGAAGAGCACACGTCT-3'

**Table S5.** Summary of data filtration analysis. Notes: (1) Sample: Sample id. (2) total_reads: Total sequenced reads. (3) N% > 10%: Percentage of reads with N > 10%. (4) low quality: Percentage of low quality reads. (5) 5_adapter_contamine: Percentage of reads with 5'adapter contaminants. (6) 3_adapter_null or insert_null: Percentage of reads with 3'adapter null or insert null. (7) with polyA/T/G/C: Percentage of reads with polyA/T/G/C. (8) clean reads: Total clean reads and its

percentage accounted for raw reads.

| Sample | total_reads | N% > 10% | low quality | 5_adapter_contamine | 3_adapter_null or insert_null | with polyA/T/G/C | clean reads |
|---|---|---|---|---|---|---|---|
| A1 | 21790669 (100.00%) | 59 (0.00%) | 0 (0.00%) | 761708 (3.50%) | 2219042 (10.18%) | 28197 (0.13%) | 18781663 (86.19%) |
| A2 | 25886953 (100.00%) | 61 (0.00%) | 0 (0.00%) | 255027 (0.99%) | 1662315 (6.42%) | 52193 (0.20%) | 23917357 (92.39%) |
| A3 | 23641594 (100.00%) | 69 (0.00%) | 0 (0.00%) | 247157 (1.05%) | 1556997 (6.59%) | 60853 (0.26%) | 21776518 (92.11%) |
| B1 | 19895748 (100.00%) | 42 (0.00%) | 0 (0.00%) | 97052 (0.49%) | 434381 (2.18%) | 36555 (0.18%) | 19327718 (97.14%) |
| B2 | 22853374 (100.00%) | 59 (0.00%) | 0 (0.00%) | 90994 (0.40%) | 562325 (2.46%) | 39324 (0.17%) | 22160672 (96.97%) |
| B3 | 24302422 (100.00%) | 54 (0.00%) | 0 (0.00%) | 80419 (0.33%) | 2037163 (8.38%) | 33045 (0.14%) | 22151741 (91.15%) |
| C1 | 25483670 (100.00%) | 56 (0.00%) | 0 (0.00%) | 538351 (2.11%) | 1220303 (4.79%) | 42979 (0.17%) | 23681981 (92.93%) |
| C2 | 25579372 (100.00%) | 8 (0.00%) | 0 (0.00%) | 340224 (1.33%) | 976728 (3.82%) | 33767 (0.13%) | 24228645 (94.72%) |
| C3 | 25790991 (100.00%) | 70 (0.00%) | 0 (0.00%) | 332511 (1.29%) | 890245 (3.45%) | 22901 (0.09%) | 24545264 (95.17%) |
| D1 | 22694237 (100.00%) | 13 (0.00%) | 0 (0.00%) | 1704979 (7.51%) | 1585156 (6.98%) | 18463 (0.08%) | 19385626 (85.42%) |
| D2 | 19709676 (100.00%) | 54 (0.00%) | 0 (0.00%) | 1276030 (6.47%) | 3701706 (18.78%) | 20236 (0.10%) | 14711650 (74.64%) |
| D3 | 19975378 (100.00%) | 45 (0.00%) | 0 (0.00%) | 3467416 (17.36%) | 6380546 (31.94%) | 16709 (0.08%) | 10110662 (50.62%) |

**Table S6 (separate file).** Raw data of proliferation levels of CD4+ T cells through Mixed Lymphocyte Reaction. Allogenic T cells have been stimulated with 3 different concentrations of dendritic cells previously incubated with 2 different dilutions of yeast EVs (A = dil 1:20, B = dil 1:200) for 4 or 24 hours. The T cells proliferation has been evaluated as counts per million of incorporated tritiated thymidine with a Microbeta 1450 Trimux counter (Wallac).
